# Gender and urban–rural difference in anthropometric indices predicting dyslipidemia in Chinese primary school children: a cross-sectional study

**DOI:** 10.1186/s12944-016-0255-y

**Published:** 2016-04-30

**Authors:** Wei Zheng, Ai Zhao, Yong Xue, Yingdong Zheng, Yun Chen, Zhishen Mu, Peiyu Wang, Yumei Zhang

**Affiliations:** Department of Social Medicine and Health Education, School of Public Health, Peking University Health Science Center, Beijing, China; CAS Key Laboratory of Pathogenic Microbiology and Immunology, Institute of Microbiology, Chinese Academy of Science, Beijing, China; Department of Epidemiology and Biostatistics, School of Public Health, Peking University Health Science Center, Beijing, China; Department of Nutrition and Food Hygiene, School of Public Health, Peking University Health Science Center, Xueyuan Road 38, Haidian District, 100191 Beijing, China; Dairy Research Institute, Inner Mongolia Mengniu Dairy (Group) Co. Ltd., Inner Mongolia, China; Beijing Key Laboratory of Toxicological Research and Risk Assessment for Food Safety, Beijing, China

**Keywords:** Serum lipid, Dyslipidemia, Body mass index, Waist-hip ratio, Mid-upper arm height ratio

## Abstract

**Background:**

Childhood dyslipidemia is a critical factor of lifelong health. Therefore, screening and controlling dyslipidemia from childhood is a practical healthy strategy. However, few studies have examined the performance of anthropometric predictors of dyslipidemia in Chinese children, let alone the potential gender and urban–rural disparity. Thus, we evaluated anthropometric indices predicting dyslipidemia by genders and living areas in Chinese children.

**Methods:**

Data were from a health and nutrition survey conducted in seven urban areas and two rural areas in China between 2011 and 2012. The serum lipid levels of the participants were compared between genders and living areas. The body mass index z-score (BMI z-score), waist-hip ratio (WHR), waist-height ratio (WHtR), and mid-upper arm height ratio (MaHtR) were used as predictors. The receiver operating characteristic (ROC) analysis was performed to investigate the ability of anthropometric indices predicting dyslipidemia.

**Results:**

A total of 773 participants (average age = 9.3 ± 1.7 y) were included. The prevalence of dyslipidemia was 10.9 %. Anthropometric indices were all significantly related to blood lipid profiles in boys after adjustment for age. The areas under the ROC curves (ACUs) were significantly larger than 0.5 in boys (ranged between 0.66–0.73), and were larger in rural boys (ranged between 0.68 and 0.94). MaHtR and WHR were associated with the highest specificity (93.8 %) and highest sensitivity (100 %), respectively.

**Conclusion:**

Using anthropometric indices, screening for dyslipidemia may be more appropriate in boys than in girls in China, especially in rural boys. The BMI z-score, WHR, WHtR, and MaHtR were all significantly associated with dyslipidemia in boys; using WHR and MaHtR as indicators achieved the highest sensitivity and specificity, respectively.

## Background

Cardiovascular disease is a global health crisis, ranked first in all causes of death in the world [1]. In China, cardiovascular disease accounts for about one-third of total mortality and costs U.S. $16 billion every year [2]. Dyslipidemia is one of the major risk factors. According to the World Health Report 2002, dyslipidemia contributed to more than half of cardiovascular disease cases [1]. It is worth noting that the prevalence of dyslipidemia increased rapidly and showed a younger-age trend during recent decades [3]. A large scale study conducted in six Chinese cities in 2010 by Zhu et al. indicated a dramatic increase in serum lipid levels in children aged 7–16 compared to results of the 2002 China Nationwide Nutrition and Health Survey [4, 5].

Meanwhile, dyslipidemia is modifiable and can be prevented or controlled through the consumption of a healthy diet and adequate physical activity from early life [3]. Accordingly, screening and controlling dyslipidemia from childhood is a practical health strategy to prevent death from cardiovascular diseases. To achieve this, estimating one’s risk of having dyslipidemia via quick, safe, inexpensive, and noninvasive procedures is critically important.

It is generally considered that excess body fat is closely related to blood lipids [6]. Therefore, a series of anthropometric indices, which were developed to evaluate body fat distribution, were evaluated regarding whether they are suitable for assessing blood lipid levels. Body mass index (BMI) is the most commonly used index, which can reflect total body composition [7]. Waist circumference (WC) serves as an important index to reflect central adiposity and is considered to be more strongly associated with certain risk factors of cardiovascular disease compared to BMI [8]. Waist-hip ratio (WHR) and waist-height ratio (WHtR) are newly emerged indices, which were suggested to be applied to children because their cut-off values are independent of age or gender [9, 10]. Mid-upper arm circumference (MUAC), the indicator best suited for screening and case detection of malnutrition in the community [11], was recently found to show an excellent agreement with BMI in prepubertal children [12]. In consideration of the close relation between BMI and blood lipid level, we speculate that it might become another alternative index predicting dyslipidemia.

On the other hand, it is widely believed that for school-age children who are experiencing dramatic changes in physical growth, the anthropometric indices used for the assessment of blood lipid levels are distinct to adults. Nevertheless, studies evaluating anthropometric indices identifying those with a high risk of dyslipidemia in children are still limited in China. In addition, in China there are significant differences between urban and rural areas. We speculated that anthropometric indices predicting dyslipidemia would be different between genders and living areas in China. Therefore, this study aimed to evaluate the differences in anthropometric indices predicting dyslipidemia between genders and living areas in Chinese school-age children.

## Methods

### Participants

Data were from a health and nutrition survey carried out between Nov. 2011 and Apr. 2012 in China. The participants were selected by a multistage cluster sampling strategy. In the first stage, seven urban areas (Beijing, Guangzhou, Chengdu, Shenyang, Suzhou, Lanzhou, and Zhengzhou city) and two rural areas (located separately in a plain area and a mountain area in Hebei province) were selected in consideration of geography and economic level. In each of the nine areas, one primary school located in the average economic level area that agreed to participate the study was included. Two classes in different grades in participant schools were randomly selected. Inclusion and exclusion criteria were as follows: All eligible children in the selected classes were included in the study. Children with reported birth defects (including congenital heart disease, hydrocephalus, and deformity at birth), infantile paralysis and thalassemia, or acute health problems (including common cold and diarrhea) at the time of survey were excluded from the study.

This study was approved by the Ethical Committee of the Health Science Center at Peking University (NO.IRB00001052-11042). Written informed consent forms were obtained from the legal guardians of all participants. Most of them were from the parents, and a very few of them were from the grandparents.

### Blood lipid profiles

Blood samples were obtained from the participants after overnight fasting. Total cholesterol (TC), triglycerides (TG), low density lipoprotein-cholesterol (LDL-C), and high density lipoprotein-cholesterol (HDL-C) were assayed by enzymatic methods using an autoanalyzer (Modular P-800; Roche, Switzerland). Concentration of LDL-C was calculated from the Friedewald equation (LDL-C = TC- (HDL-C + TG/5)).

The definition of *dyslipidemia* was taken from the National Cholesterol Education Program (NCEP) [13] and “Experts Consensus for Prevention and Treatment of Dyslipidemia in Children and Adolescents” in China [14]. The cut-off of each type of dyslipidemia was defined as follows: TC ≥ 200 mg/dL (5.172 mmol/L), LDL-C ≥ 130 mg/dL (3.3618 mmol/L), TG ≥ 150 mg/dL (1.6935 mmol/L), and HDL-C ≤ 35 mg/dL (0.9051 mmol/L). The prevalence of dyslipidemia was defined to be the occurrence of any or all types of dyslipidemia.

### Anthropometric measurements

The children’s anthropometric characteristics were measured by trained researchers in a comfortable examination area with the children wearing minimal clothing. Height was measured accurate to 0.1 cm, and weight was measured accurate to 0.1 kg. WC was measured at 2 cm above the umbilicus. HC was measured at maximal protrusion of the buttocks. MUAC was measured midway between the acromion and olecranon on the non-dominant arm. All of these indices were accurate to 0.1 cm. BMI was calculated as weight/height^2^ (kg/m^2^). The BMI z-score was calculated according to the criteria of the World Health Organization. WHR was calculated as WC/HC, WHtR was calculated as WC/height, and mid-upper arm height ratio (MaHtR) was calculated as MUAC/height.

### Other measurements

Questionnaire surveys was administered to the legal guardians of the participants to collect related information, including gender, age, living area, family income per capita, total physical activity time per week, energy intake per day, and nutraceutical use.

### Statistical methods

The serum lipid profiles between different groups of participants were compared using Student’s *t*-test. Partial correlations between anthropometric measurements and serum lipid profiles adjusted for age were examined using Spearman’s correlation test.

The receiver operating characteristic (ROC) analysis was performed to investigate the diagnostic ability of anthropometric indices in identifying the risk of dyslipidemia. The ROC curve is a plot of sensitivity versus 1-specificity. The area under the ROC curve (AUC) was estimated to measure the overall performance of each anthropometric index predicting risk of dyslipidemia. Optimal cut-off points for each anthropometric index were determined using the maximum value of Youden’s index (i.e., sensitivity + specificity - 1), corresponding to the nearest point of the ROC curve to the upper left-hand corner.

The Bonferroni test was used for comparison of AUCs using the BMI z-score, WHR, WHtR, and MaHtR as predictors. The crude odds ratio (OR) and OR adjusted for age, energy intake, total physical activity, nutraceutical use, living area, and family income were calculated to assess the association between anthropometric indices and dyslipidemia. OR was estimated by every increase of approximately one standard deviation of each anthropometric index.

All statistical analyses were conducted using SAS version 9.3 (SAS Institute Inc., Cary, NC, USA).

## Results

A total of 932 school-age children participated in the health and nutrition survey. Of these participants, 773 with both anthropometric and blood lipid profile data were included in our analysis. No significant differences were observed in the basic characteristics between children with and without complete information (data not shown). Their average age was 9.3 ± 1.7 years old. Among these children, 2.6 % were identified with high TC, 8.3 % with TG, 1.0 % with high LDL-C, and 2.4 % with low HDL-C. Prevalence of any or all types of dyslipidemia was 10.9 %, 12.4 % in boys and 8.5 % in girls. Differences in serum lipid profiles between genders and living areas are indicated in Table 1. Children in rural areas showed lower TC, lower HDL-C and lower LDL-C in comparison with children in urban areas. No significant differences between genders were identified.

**Table 1:** Serum lipid profiles (mmol/L) in Chinese primary school children by gender and by living area

	Urban area				*p*-value * (between genders)	Rural area				*p*-value * (between genders)	*p*-value * (between urban and rural areas)
	Boys		Girls			Boys		Girls			
	Mean	SD	Mean	SD		Mean	SD	Mean	SD		
n	311		272			88		102			
TC	3.96	0.62	3.96	0.63	0.9	3.43	0.54	3.58	0.69	0.09	<0.0001
TG	0.99	0.52	1.04	0.60	0.3	0.92	0.64	0.95	0.47	0.7	0.1
HDL-C	1.49	0.36	1.46	0.33	0.2	1.40	0.26	1.41	0.27	0.7	0.006
LDL-C	1.94	0.52	1.94	0.52	1	1.64	0.40	1.74	0.60	0.2	<0.0001

*TC* total cholesterol, *TG* triglyceride, *HDL-C* high density lipoprotein cholesterol, *LDL-C* low density lipoprotein cholesterol

*p-value was calculated by Student’s *t*-test.

Results from the Spearman’s correlation test are shown in Table 2. In boys, the BMI z-score, WHR, WHtR, and MaHtR were all positively related to TC, TG, and LDL-C and inversely related to HDL-C, after adjustment for age. However, correlation between few anthropometric indices and lipid indicators was observed in girls.

**Table 2:** Partial correlation coefficients between anthropometric measurements and serum lipid profiles^a^

	TC Coefficient (*p*-value)	TG Coefficient (*p*-value)	HDL-C Coefficient (*p*-value)	LDL-C Coefficient (*p*-value)
Boys				
BMI z-score	0.22 (<0.0001)	0.22 (<0.0001)	−0.12 (0.01)	0.21 (<0.0001)
WHR	0.15 (0.002)	0.26 (<0.0001)	−0.18 (0.0003)	0.14 (0.007)
WHtR	0.21 (<0.0001)	0.31 (<0.0001)	−0.18 (0.0003)	0.19 (0.0002)
MaHtR	0.27 (<0.0001)	0.26 (<0.0001)	−0.07 (0.1)	0.23 (<0.0001)
Girls				
BMI z-score	0.10 (0.06)	0.11 (0.04)	−0.10 (0.05)	0.11 (0.04)
WHR	−0.05 (0.4)	0.10 (0.047)	−0.13 (0.02)	−0.03 (0.5)
WHtR	0.01 (0.8)	0.10 (0.07)	−0.15 (0.003)	0.03 (0.5)
MaHtR	0.11 (0.03)	0.08 (0.1)	−0.006 (0.9)	0.08 (0.1)

*TC* total cholesterol, *TG* triglyceride, *HDL-C* high density lipoprotein cholesterol, *LDL-C* low density lipoprotein cholesterol, *BMI* body mass index, *WHR* waist-hip ratio, *WHtR* waist-height ratio, *MaHtR* mid-upper arm height ratio

^a^Adjusted for age

Table 3 shows the results of the ROC analysis in boys. All AUCs were significantly larger than 0.5 except for the BMI z-score in rural boys. AUC was significantly larger in WHtR than in the BMI z-score predicting dyslipidemia (*p* = 0.03). No difference in AUCs between other anthropometric indices were identified. ACUs in rural boys are larger than in urban boys. In accordance with the ACU, the maximum value of Youden’s index was larger in rural boys. Sensitivity and specificity corresponding to the optimal cut-off points of each anthropometric index are also shown in Table 3. In general, using MaHtR as the indicator achieved the highest specificity while WHR was associated with the highest sensitivity. Results of the ORs and adjusted ORs indicated that an increase in all anthropometric indicators was significantly associated with increased risk of dyslipidemia. OR was larger in rural boys.

**Table 3:** AUCs, cut-off points and sensitivity/specificity of anthropometric measurements predicting dyslipidemia in Chinese boys (2011–2012)

	AUCs (95 % CI)	Cut-off points	Sensitivity (%)	Specificity (%)	OR^a^	Adjusted OR^b^
Total						
BMI z-score	0.66 (0.57–0.75)	0.973	59.6	73.2	1.88 (1.38–2.61)	2.08 (1.36–3.25)
WHR	0.73 (0.66–0.80)	0.862	70.2	70.3	1.50 (1.21–1.89)	1.81 (1.34–2.48)
WHtR	0.72 (0.65–0.80)	0.473	59.6	76.6	1.89 (1.46–2.47)	2.20 (1.52–3.27)
MaHtR	0.67 (0.59–0.76)	0.159	57.4	77.5	1.76 (1.32–2.37)	2.10 (1.37–3.28)
Urban area						
BMI z-score	0.64 (0.54–0.74)	1.309	57.5	74.2	1.85 (1.28–2.71)	1.91 (1.12–3.36)
WHR	0.68 (0.60–0.76)	0.836	82.5	50.7	1.34 (1.08–1.69)	1.47 (1.04–2.09)
WHtR	0.69 (0.60–0.77)	0.473	60.0	72.0	1.74 (1.31–2.32)	1.87 (1.20–3.00)
MaHtR	0.65 (0.55–0.75)	0.159	62.5	72.0	1.62 (1.16–2.27)	1.70 (0.99–3.01)
Rural area						
BMI z-score	0.68 (0.40–0.97)	−0.422	71.4	76.3	2.18 (0.98–5.03)	2.64 (1.07–8.00)
WHR	0.94 (0.88–1.00)	0.864	100	81.3	5.90 (2.29–21.68)	5.27 (1.74–26.71)
WHtR	0.87 (0.73–1.00)	0.459	85.7	85.0	3.13 (1.47–8.00)	2.78 (1.20–9.11)
MaHtR	0.77 (0.53–1.00)	0.145	57.1	93.8	3.47 (1.45–8.96)	4.43 (1.45–22.43)

*AUC* area under the receiver operating characteristic curve, *CI* confidence interval, *OR* odds ratio, *WC* waist circumference, *BMI* body mass index, *WHR* waist-hip ratio, *WHtR* waist-height ratio, *MaHtR* mid-upper arm height ratio

^a^OR was calculated by every increase of approximately one standard deviation of anthropometric measurements (1.5 for BMI z-score, 0.05 for WHR and WHtR, 0.02 for MHR)

^b^Adjusted for age, energy intake, total physical activity, nutraceutical use, living area, and family income

## Discussion

This study indicated that serum lipid levels were higher in urban Chinese primary school children than in rural children. The average levels of lipid profiles in urban children and rural children in this study were approximately 18 % and 10 % higher than the results in children aged 7–12 years old in the 2002 national survey. This difference suggested a secular increase in blood lipid levels. On the other hand, serum lipid levels in the urban area in this study were almost the same as the results in Zhu et al.’s study in 2010.

This study also assessed the predicting ability of different anthropometric indices in dyslipidemia. A close relation between anthropometric indices and blood lipid profiles was identified in boys but not in girls. Results from the ROC analysis in boys showed a >0.5 AUCs in all anthropometric indices.

ACUs in rural boys are larger than in urban boys. MaHtR was related to the highest specificity while WHR was associated with the highest sensitivity in predicting dyslipidemia.

Numerous studies have been conducted to explore and confirm the use of anthropometric indices in predicting cardiovascular diseases. BMI, WC, and WHR are the most commonly used indicators. For children who are experiencing rapid physical growth, age specific BMI, BMI z-score, age-specific WC, and WHtR are widely used. A study by Sung et al. indicated that age-specific BMI and WC were associated with cardiovascular risk factors including fasting insulin, TG, HDL, and LDL in Chinese children [15]. Moreno et al. indicated that age-specific WC was superior to BMI in predicting metabolic syndrome in children [16]. Another study by Kuba et al. suggested that WHtR and BMI z-score were both sensitive indices in screening for metabolic risk factors in 6–10-year-old children [9]. All of the aforementioned indices were confirmed to be associated with a variety of cardiovascular risk factors. These associations were consistent with the results in this study in boys.

Other easy to apply indices for screening purpose are still being explored. MUAC is one of the candidate indicators. An excellent agreement between MUAC and BMI was observed in a previous study by Ayatollahi et al. The authors used age-specific MUAC in consideration of overall physical growth during the pre-pubertal stage. In this study, we used MaHtR as an indicator for two reasons. Firstly, MaHtR took standing height into consideration. Even in children at the same age, physical growth may vary greatly. The influence of height should not be ignored when measuring fat deposition using MUAC. Secondly, calculating MaHtR is a simpler procedure under certain conditions in a practical application. Meanwhile, due to the limited sample size, MUAC for age could not be used for analysis in this study. In this study, using MaHtR as an indicator may achieve the highest specificity. Therefore, MaHtR may be combined with WHR for screening the risk of dyslipidemia in boys.

On the other hand, this study identified both gender and urban–rural disparities in the predicting ability of the examined indices. In China, there is a large urban–rural disparity in many aspects, including social economic status [17], the health-care system and its utilization [18, 19], diet pattern, nutrition status, and physical development [20]. In this study, better TC and LDL-C levels were observed in rural children while better HDL-C levels were identified in urban children. The performance of anthropometric indices in screening for dyslipidemia was better in rural boys. These results suggested that in rural boys fat deposition may be a major risk factor of dyslipidemia while in urban boys other factors may contribute largely to blood lipid level. Thus, when applying anthropometric predictors, it is better to take other influencing factors into account.

In this study, only a weak association between certain anthropometric indices and blood lipid profiles was identified in girls. Many studies did not refer to gender differences in their analysis [8, 9, 21, 22]. Some other studies that were stratified by gender showed a gender disparity, but most of them did not discuss this issue. They used more complicated indicators such as a set of cardiovascular disease risk factors, including blood pressure, fasting blood glucose level, and blood lipid level. The combination of indicators obscured the gender disparity in dyslipidemia [15, 23]. One previous study carried out in Shanghai, China indicated similar gender differences to this study [7]. They only reported a reduction in HDL-C and an increase in TG with elevated BMI in girls. Another study by Savva et al. indicated that LDL and TG but not TC were associated with anthropometric indices [23]. A study conducted in Hong Kong indicated that BMI was not related to LDL in girls [15]. In these above-mentioned studies, anthropometric indices were significantly associated with all of the blood lipid profiles in boys.

A possible explanation for the gender disparity is related to the sexual dimorphism of fat distribution and hormone levels. Wells [24] indicated that sexual dimorphism in body composition is evident since the fetal stage, throughout childhood, and then enlarged from the pubertal stage. A study by Nedungadi and Clegg demonstrated [25] that females had more subcutaneous fat whereas male had more visceral fat, which indicated a greater risk for dyslipidemia. In addition, studies have clarified that leptin and adiponectin levels (which were considered to be cardiovascular-protective factors because of their lipid metabolic regulation effect [26, 27]) are higher in girls than in boys after adjustment for body fat content from birth [28–30]. Accordingly, association between anthropometric measurements and blood lipid profiles is weaker in girls than in boys. Thus, using anthropometric indices as predictors of dyslipidemia in girls may be not as accurate as in boys. In addition, girls entered puberty earlier than boys. Changes in body composition during pubertal development may influence the predicting ability of anthropometric indices.

Moreover, this study indicated a strong positive association between anthropometric indices and the risk of dyslipidemia after adjustment for factors influencing dyslipidemia. The adjusted factors included age, energy intake, total physical activity, nutraceutical use [31], living area, and family income.

There are several limitations that need to be addressed. Firstly, although we investigated children from nine areas all over China in view of geography and economic level, this is not a representative sample of Chinese children. Moreover, in each area, only one school participated in the study. The sample size was limited in this study, especially in the subgroup analysis. Therefore, further large scale studies are needed to confirm the gender and urban–rural disparities. Secondly, we did not investigate the health conditions of participants’ parents, which may be critical to childhood dyslipidemia. Thirdly, we did not investigate other cardiovascular risk factors such as blood pressure and fasting blood glucose. Therefore, the application of this study was confined to blood lipid disorders. Fourth, we did not adjust for children’s pubertal development status. Further studies including pubertal indicators might help improve the predicting ability of anthropometric indices.

## Conclusion

In conclusion, this study demonstrated that serum lipid levels in Chinese children increased dramatically since 2002, and a significant difference between urban and rural area was observed. The BMI z-score, WHR, WHtR, and MaHtR were all significantly associated with dyslipidemia in boys; using WHR and MaHtR as indicators achieved the highest sensitivity and specificity, respectively. In addition, evident gender and urban–rural disparities in the anthropometric predictors used in screening for dyslipidemia were observed. Therefore, using anthropometric indices to assess the risk of dyslipidemia may be more appropriate in boys than in girls in China. Moreover, anthropometric indices might be better predictors in rural boys. However, in consideration of the relatively small sample size in this study, we should be cautious in applying anthropometric indices as blood lipid level predictors to practice in China before further confirmation.
