# Erratum to: Gender and urban–rural difference in anthropometric indices predicting dyslipidemia in Chinese primary school children: a cross-sectional study

**DOI:** 10.1186/s12944-016-0299-z

**Published:** 2016-08-12

**Authors:** Wei Zheng, Ai Zhao, Yong Xue, Yingdong Zheng, Yun Chen, Zhishen Mu, Peiyu Wang, Yumei Zhang

**Affiliations:** 1 Department of Social Medicine and Health Education, School of Public Health, Peking University Health Science Center, Beijing, China; 2 CAS Key Laboratory of Pathogenic Microbiology and Immunology, Institute of Microbiology, Chinese Academy of Science, Beijing, China; 3 Department of Epidemiology and Biostatistics, School of Public Health, Peking University Health Science Center, Beijing, China; 4 Department of Nutrition and Food Hygiene, School of Public Health, Peking University Health Science Center, Xueyuan Road 38, Haidian District, Beijing, 100191 China; 5 Dairy Research Institute, Inner Mongolia Mengniu Dairy (Group) Co. Ltd., Inner Mongolia, China; 6 Beijing Key Laboratory of Toxicological Research and Risk Assessment for Food Safety, Beijing, China

## Erratum

After publication of the original article [1], it came to the authors’ attention that a funding number in the ‘Financial support’ section had been incorrectly specified. The correct funding number of Key Projects of Beijing Science & Technology is D141100004814002, not Z1411000048140.
